# Genomic selection in United States dairy cattle

**DOI:** 10.3389/fgene.2022.994466

**Published:** 2022-09-09

**Authors:** George R. Wiggans, José A. Carrillo

**Affiliations:** Council on Dairy Cattle Breeding, Bowie, MD, United States

**Keywords:** genomic selection, dairy cattle, genetic gain, genetic-economic index, breed composition, ancestor discovery, haplotype, recessive discovery

## Abstract

The genomic selection program for dairy cattle in the United States has doubled the rate of genetic gain. Since 2010, the average annual increase in net merit has been $85 compared to $40 during the previous 5 years. The number of genotypes has been rapidly increasing both domestically and internationally and reached over 6.5 million in 2022 with 1,134,593 submitted in 2021. Evaluations are calculated for over 50 traits. Feed efficiency (residual feed intake), heifer and cow livability, age at first calving, six health traits, and gestation length have been added in recent years to represent the economic value of selection candidates more accurately; work is underway to develop evaluations for hoof health. Evaluations of animals with newly submitted genotypes are calculated weekly. In April 2019, evaluations were extended to crossbreds; to support that effort, evaluations are initially calculated on an all-breed base and then blended by an estimated breed composition. For animals that are less than 90% of one breed, the evaluation is calculated by weighting contributions of each of the five major dairy breeds evaluated (Ayrshire, Brown Swiss, Guernsey, Holstein, and Jersey) by the breed proportion. Nearly 200,000 animals received blended evaluations in July 2022. Pedigree is augmented by using haplotype matching to discover maternal grandsires and great-grandsires. Haplotype analysis is also used to discover undesirable recessive conditions. In many cases, the causative variant has been identified, and results from a gene test or inclusion on a genotyping chip improves the accuracy of those determinations for the current 27 conditions reported. Recently discovered recessive conditions include neuropathy with splayed forelimbs in Jerseys, early embryonic death in Holsteins, and curly calves in Ayrshires. Techniques have been developed to support rapid searches for parent-progeny relationships and identical genotypes among all likely genotypes, which substantially reduces processing time. Work continues on using sequence data to discover additional informative single nucleotide polymorphisms and to incorporate those previously discovered. Adoption of genotyping by sequencing is expected to improve flexibility of marker selection. The success of the Council on Dairy Cattle Breeding in conducting the genetic evaluation program is the result of close cooperation with industry and research groups, including the United States Department of Agriculture, breed associations, genotyping laboratories, and artificial-insemination organizations.

## 1 Introduction

Genomic selection has revolutionized dairy cattle breeding by doubling the rate of genetic gain primarily through halving the generation interval. In the United States, the Council on Dairy Cattle Breeding (CDCB) conducts a genetic evaluation program that includes genotypes from all over the world. The number of genotypes in the collection has been rapidly increasing and reached 6.6 million in August 2022 with 1,134,593 submitted in 2021 (Council on Dairy Cattle Breeding, 2022b). Continuous refinement of the program involves incorporating research results to improve accuracy, exploiting technological advances, and adapting to changes in the industry. Changes include extending evaluations to crossbreds, increasing the frequency of evaluation, revising the set of genotype markers used, adding evaluations for health, reproduction and feed efficiency traits, updating genetic indexes to improve ranking based on economic value, detecting additional deleterious genetic factors, augmenting pedigree by discovering ancestors, and providing breed composition information. This article is an update and expansion of Wiggans (2017).

## 2 Characteristics of genomic evaluation system in the United States

**FIGURE 1:**
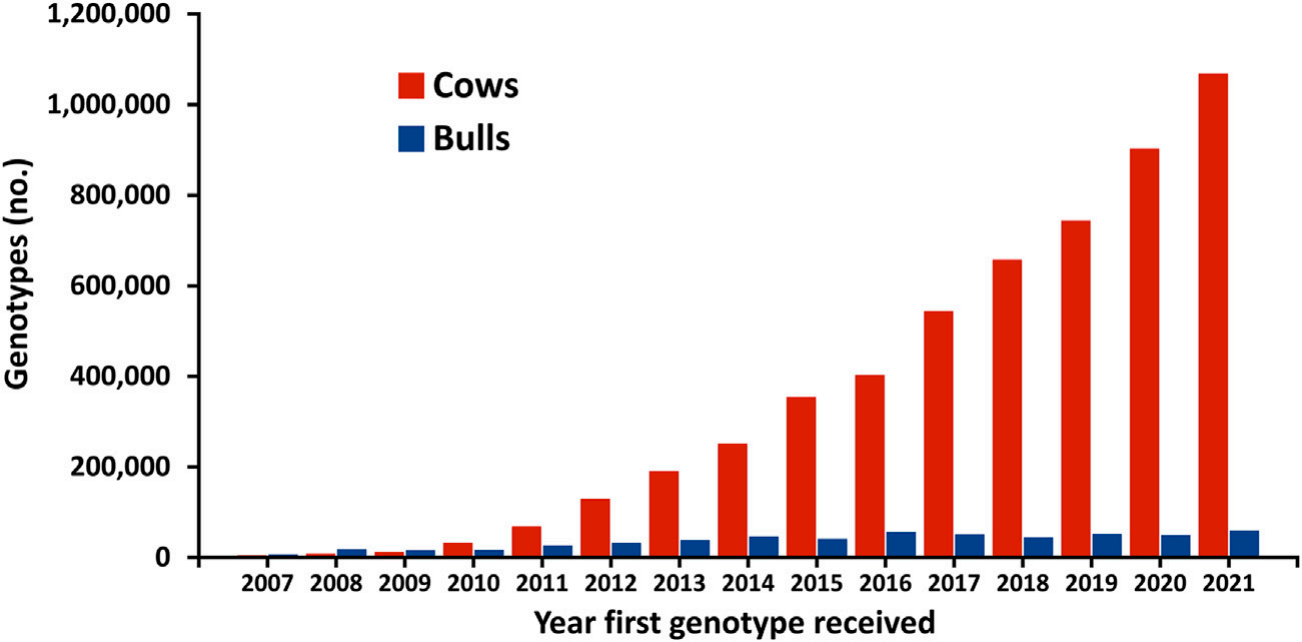
Number of dairy cattle genotypes submitted in the United States by year that first genotype was received.

**FIGURE 2:**
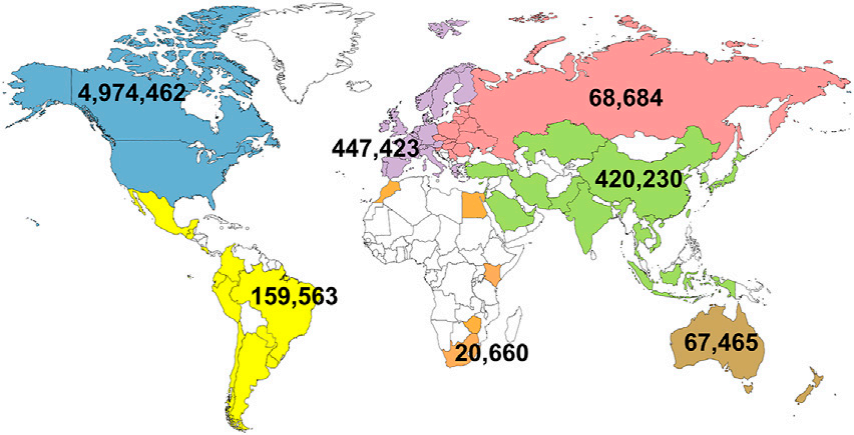
Total number of genotypes that have been submitted in the United States by global region as of June 2022.

**FIGURE 3:**
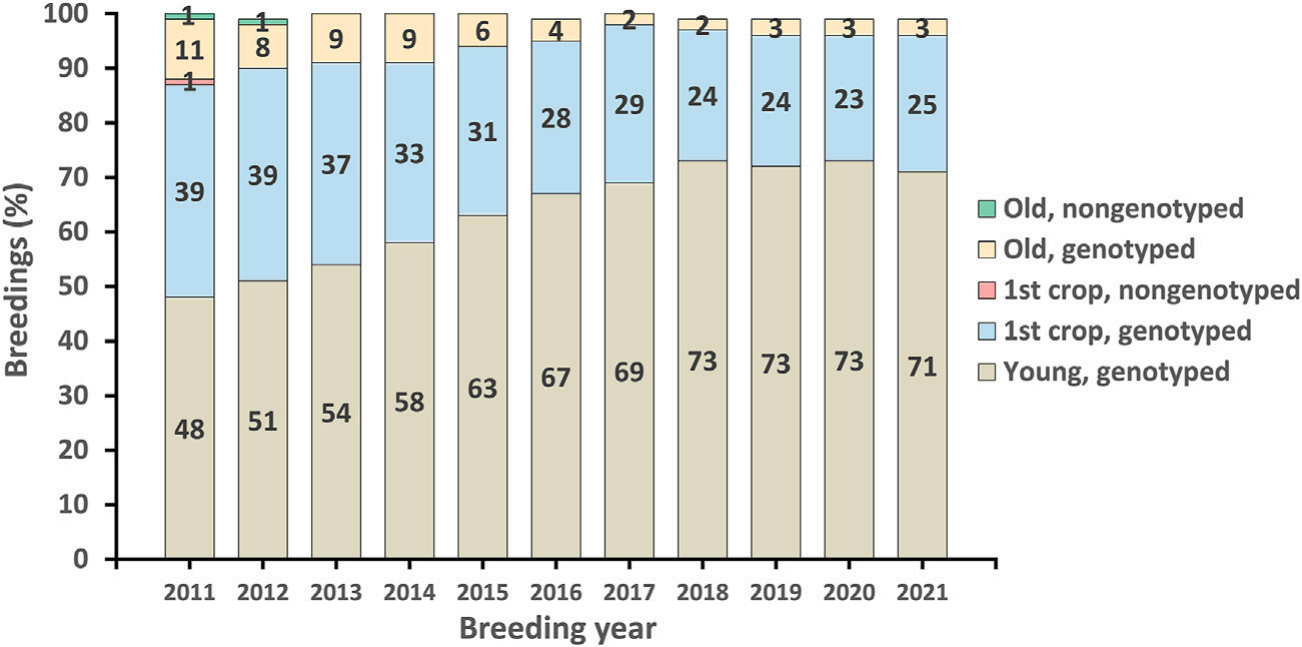
Genomic profile of Holstein service sires used for artificial-insemination breeding in the United States since 2011.

The first official genomic evaluations were released in January 2009 for Holsteins and Jerseys. Figure 1 shows the growth in number of genotypes submitted (excluding withdrawn) by year. In 2008 and 2009, more genotypes for bulls were received than for cows, however, in later years, the number of bull genotypes received has been nearly constant while the number of genotypes of females received increased rapidly. Figure 2 shows the number of genotypes submitted as of June 2022 by global region. Genomic evaluations were rapidly accepted by the dairy industry as the basis for selecting service sires. In just a few years, the majority of breedings were to bulls with only genomic evaluations (Figure 3).

**FIGURE 4:**
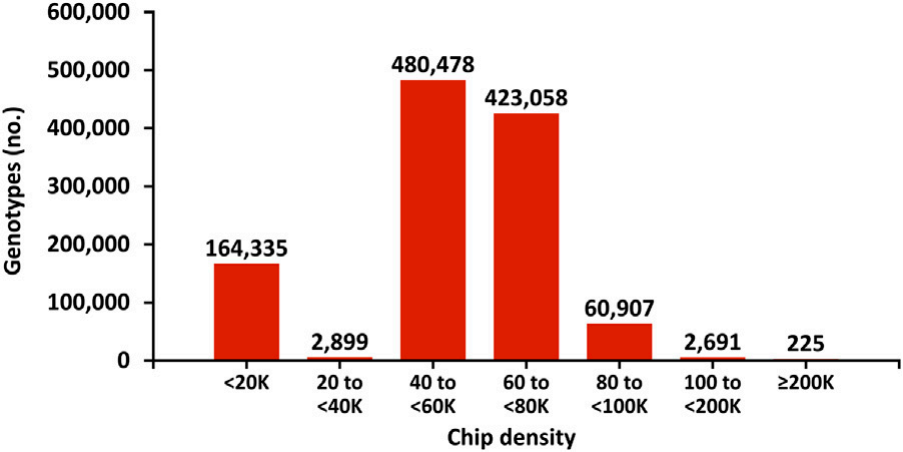
Marker density of genotyping chips for genotypes received in 2021 in the United States.

The genotyping chips used for dairy cattle also have evolved. To reduce the cost of genotyping, chips with fewer single nucleotide polymorphism (SNP) markers were introduced after the initial 50 K chip (54,001 SNPs). As technology advanced, higher density chips were offered. Typically, bulls that are marketed, have two genotypes: the first to determine if they rank high enough to be marketable and the second with higher density to maximize the accuracy of their evaluation by minimizing imputation errors. Figure 4 shows the distribution of chip densities for genotypes received in 2021.

**FIGURE 5:**
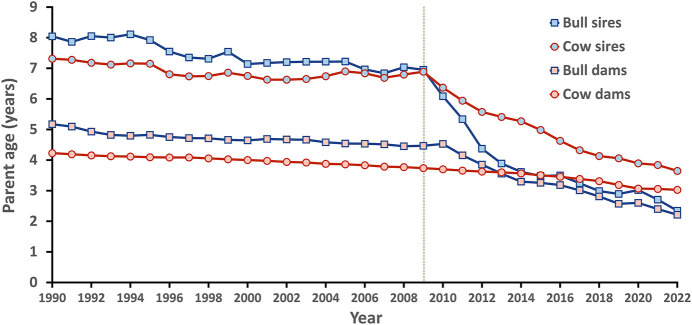
Generation intervals for Holsteins in the United States by sex and year.

Generation interval is the average age of parents when offspring are born and impacts genetic improvement. The shorter the generation interval, the faster progress can be made so long as accuracy is not compromised excessively. A few years after genomic evaluations became official in 2009, parent ages for bulls began to drop dramatically and are now near the biological minimum (Figure 5).

**FIGURE 6:**
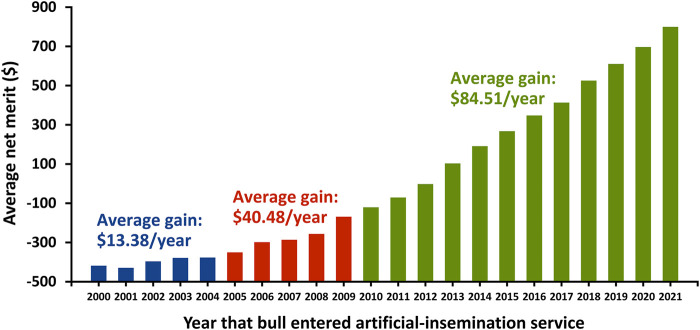
Gain in net merit for marketed Holstein bulls in the United States by entry year into artificial-insemination service.

Genomic selection has produced a large increase in genetic trend as indicated by the average net merit of marketed Holstein bulls in the United States (Figure 6). The $85 annual trend for Holstein bulls that entered artificial-insemination service since 2011 is more than double that for the period from 2005 through 2009 period ($40), which was already a substantial improvement over the $13 average gain for the period from 2000 through 2004.

### 2.1 Traits evaluated

The national genetic evaluations calculated by the CDCB currently include over 50 traits (Council on Dairy Cattle Breeding, 2022a). Production traits include milk yield, fat yield and percentage, and protein yield and percentage. Milking speed is collected only through the Brown Swiss type program so is only evaluated for that breed. Eighteen conformation (type) traits are included for non-Holstein breeds; Holstein conformation evaluations are calculated by Holstein Association USA (2022). Longevity traits include productive life, cow livability, and heifer livability (birth to first calving, added in 2020). Fertility traits include daughter pregnancy rate, cow conception rate, calving to first insemination, gestation length, and early first calving (added in 2019); male fertility is evaluated phenotypically as service-sire relative conception rate. The calving traits of dystocia (calving ease) and stillbirth rate are combined into a calving ability index. In addition to traditional evaluations for somatic cell score as a measure of mastitis resistance, evaluations for other health traits were introduced in 2018: displaced abomasum, ketosis, mastitis, metritis, milk fever (hypocalcemia), and retained placenta. In 2020, the trait feed saved was added as a measure of genetic merit for feed efficiency; it combines evaluations of body weight composite and residual feed intake (Council on Dairy Cattle Breeding, 2020). Most traits make a direct contribution to economic value. Gestation length is not included in the economic indexes; however, it is correlated with calving traits and may be useful in pasture-based systems to assist in determining calving date.

### 2.2 Genetic-economic indices

**FIGURE 7:**
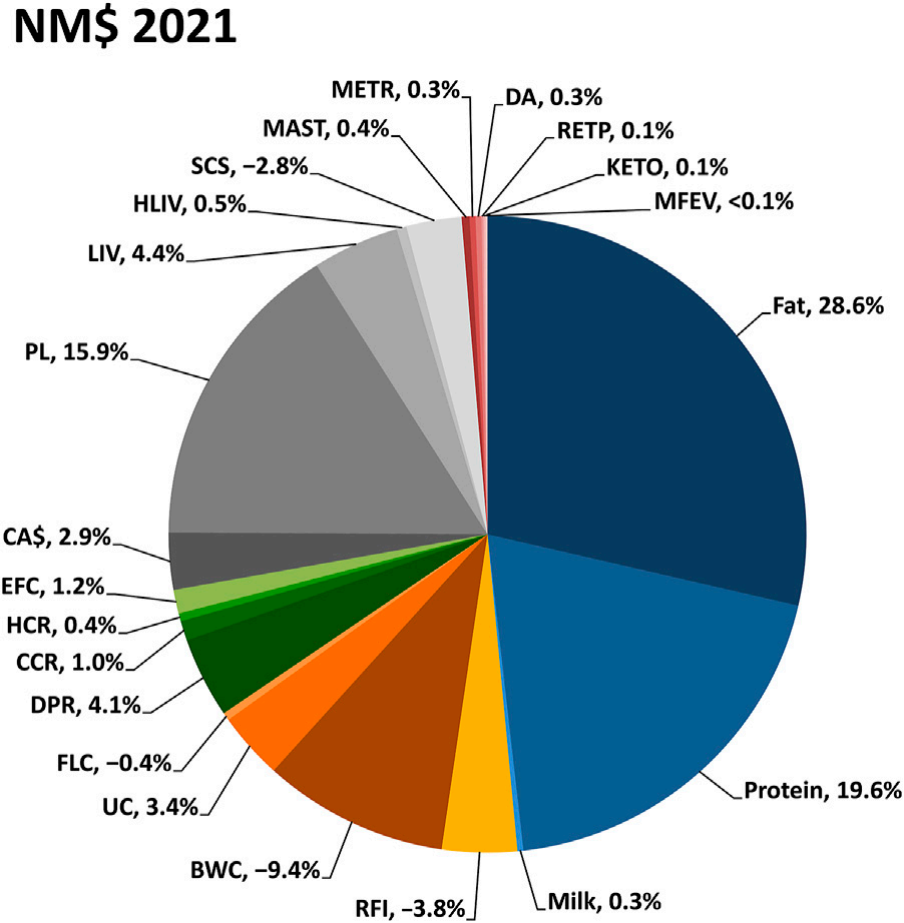
Relative emphasis on traits in the net merit (NM$) genetic-economic index revised in August 2021 by the Council on Dairy Cattle Breeding. Body weight composite, BWC; calving ability (calving ease and stillbirth rate), CA$; cow conception rate, CCR; cow livability, LIV; daughter pregnancy rate, DPR; displaced abomasum, DA; early first calving, EFC; feet-legs composite, FLC; heifer conception rate, HCR; heifer livability, HLIV; ketosis, KETO; mastitis, MAST; metritis, METR; milk fever, MFEV; productive life, PL; residual feed intake, RFI; retained placenta, RETP; somatic cell score, SCS; udder composite, UC.

Lifetime genetic-economic indices are provided to the dairy industry for net merit, fluid merit, cheese merit, and grazing merit (Vanraden et al., 2021). Those indices rank animals based on their combined genetic merit for economically important traits. Multiple indexes are provided to support selection in a range of management and milk payment schemes. The indices are updated periodically to include new traits and to reflect prices expected in the next few years. The most recent update was in August 2021 and included information for the newly evaluated traits of feed saved, heifer livability, and early first calving (Figure 7).

### 2.3 Evaluation calculation features

Genomic evaluations are based on estimation of allele substitution effects for 78,964 SNPs selected considering minor allele frequency, distribution across the genome, linkage to genes of particular interest, and reliability considering call rate and Mendelian consistency. The sum of SNP effects, called direct genomic value, is combined with an estimate of polygenetic effects and the traditional evaluation to create the genomic evaluation (Vanraden, 2008). This is called the two-step method because traditional evaluations are calculated without the genomic data, which prevents consideration of selection based on genomic information and could cause selection bias. A single-step approach has been developed to allow simultaneous consideration of the genotypes and trait observations (Legarra and Ducrocq, 2012). Adapting the one-step method to the massive US dataset is an ongoing research project.

#### 2.3.1 Estimation of breed composition

Breed composition is estimated with the same set of 78,964 SNPs used to determine the direct genomic values for other traits. The predictor population is purebred bulls, and the data are defined as one for the animal’s breed and 0 otherwise. Solutions (as percentages) are forced to add to 100, and portions of less than 2% are distributed to remaining breeds. The percentages are called breed base representation (BBR). They are used to validate the breed of the identification data for an animal and weight individual breed contribution to the evaluations of crossbreds.

#### 2.3.2 Evaluation of crossbreds

Because SNP effects differ by breed, genomic evaluations are calculated separately by breed. To provide evaluations for crossbreds, SNP effects from the individual breeds are combined. Animals with a highest BBR of less than 89.5% are evaluated as crossbreds, and their evaluations are a blend of their direct genomic values weighted by their BBRs for each breed. This blending is possible for traits that are initially evaluated on an all-breed base. Type traits and traits that are not calculated for all breeds are not blended; therefore, the animal receives an evaluation for the breed with the highest BBR. For first-generation crosses, the breed from the preferred identification determines the breed of evaluation. Nearly 200,000 animals received blended evaluations in July 2022.

### 2.4 Pedigree validation and discovery

Each genotype is compared with the genotypes of parents and then with all existing genotypes that might have a parent-progeny relationship or be identical. To limit the time required, birth date limits are imposed. If both parents are confirmed, the search is limited to genotypes of animals born no more than 5 years before. That limit is increased to 12 years for other animals. Genotypes of bulls born more than 5 years ago without progeny born in the preceding 5 years are skipped unless their genotype was added to the evaluation system within the last year. No animals with genotyped progeny are skipped. Animals with conflicting parents or discovered relationships are not evaluated until the conflicts are resolved. In general, for a conflicting pair, the genotype with the less reliable information is the one designated as not usable. If a parent is not genotyped or not confirmed, the likelihood of the grandsire is determined. If the grandsire is unlikely, the animal is not evaluated.

Discovery of maternal grandsires (MGS) and maternal great-grandsires (MGGS) is done as part of the evaluation based on haplotypes in common. An imputation process is used to create genotypes with 78,964 SNPs from incoming genotypes of various lengths. The genome is divided into intervals and maternal or paternal origin is determined. Those haplotypes are compared, and bulls are designated as discovered ancestors based on the percentage of haplotypes in common. Crossing over reduces the expected haplotypes in common to 45% for MGS and 20% for MGGS. A bull’s percentage must exceed the next highest bull’s by 15% and have a percent matches greater than 35% for MGS and 15% for MGGS to be designated as discovered. The age of the bull at the birth of the grand progeny/great grand progeny is considered. The discoveries are used to remove an unlikely grandsire designation if the discovered grandsire is the same as the pedigree grandsire. If no pedigree information on a dam or granddam was provided, the discovered MGS and MGGS are added to the pedigree. Similarly, if the connecting dam is unknown, identification data will be constructed so that a pedigree record can be created to store the MGS or MGGS information.

To speed discovery, a set of 3,552 SNPs that are present on most genotyping chips and have high call rate and good Mendelian consistency were selected. Comparisons are ordered so that checking stops after 96 or 1,000 SNPs if the percentage of conflicts exceeds that likely for a parent-progeny relationship. The discovered closely related pairs are stored using a unique genotype identification so that the identity of close relatives is not affected by genotype reassignment.

### 2.5 Mating decisions

The CDCB provides information on the genomic relationships between potential dams and currently marketed bulls. Those data report the actual portion of genetic variants (alleles) in common, in contrast to pedigree analysis, which can only give the average based on relationships. This allows avoiding inbreeding more precisely.

The CDCB also provides predictions for a number of recessive conditions so that likely carrier-to-carrier matings can be avoided even without testing an animal. The haplotypes that affect fertility are conditions discovered through genomics and can now be considered in matings. Currently, 27 conditions are reported (Cole et al., 2022). Recently added recessives include JNS (neuropathy with splayed forelimbs in Jerseys), HH6 (early embryonic death in Holsteins), and AHC (curly calves in Ayrshires).

Future mating programs may also consider the effects of dominance, which causes some sire-MGS grandsire combinations to do better than expected and others to do worse.

## 3 Methods to increase evaluation accuracy

Genomic evaluation relies on having enough DNA markers to track the segments of chromosomes associated with high performance across generations. The SNPs are used as markers because of their reliability and low cost. However, crossing over (recombination) can disrupt the associations between a marker and the causative variant, which results in a decay in the linkage between SNPs and genomic regions associated with high performance. To counteract this, new data are needed so that the SNP effects can be re-estimated to maintain or improve evaluation accuracy. Now that the cost of whole-genome sequencing has fallen and thousands of animals have been sequenced, research is focused on finding SNPs that are more closely associated with the causative genetic variants (or even the variants themselves). The closer the marker is to the causative variant, the lower the likelihood that recombination will disrupt the linkage.

### 3.1 More traits

For traditional genetic evaluations, an animal could only receive an evaluation for a trait that was observed for the animal or its offspring. Evaluation were limited to traits where large scale collection of data was possible such and milk and fat yield and type traits collected by breed associations. With genomics, evaluations can be generated for all genotyped animals if enough animals have genotypes and traditional evaluations for the trait (the reference population) to give reasonably accurate estimates of the SNP effects. Feed efficiency is an example of a small population of animals with feed intake measured providing the basis for feed saved evaluations for all genotyped animals. Efforts to collect data for more traits are ongoing. Foot health and milking speed are currently in the research phase. Mid-infrared spectroscopy of milk samples is expected to provide data related to traits of economic importance.

### 3.2 Larger reference population

Early in the development of the US genomic evaluation system, arrangements were made to share genotypes between countries to increase the size of the reference population (Wiggans et al., 2011). From the beginning, all US genotypes have been shared with Canada. For Holsteins, sharing is ongoing with Italy, the United Kingdom, Switzerland, and Germany. The reference population for Holsteins is so large that the value of adding older animals has declined; however, the addition of younger animals is still beneficial. The greatest benefit from sharing genotypes and phenotypic data may be for feed efficiency because of the high cost of data collection.

### 3.3 Better and more data

Herds with high levels of genotyping generally have fewer misidentified sires in their data that contribute to traditional evaluations. With better data, less information is lost from the extensive checks done by the CDCB to eliminate unreliable and inconsistent data. Genotypes are checked against all other genotypes to ensure they are assigned to the correct animal and that the parents are correctly identified. Data problems can include submitting the same identification number for different cows, not documenting that sexed semen was used for an insemination and reporting the transfer of an embryo to a recipient as a normal breeding. Greater knowledge of data usage should help providers better understand the importance and benefit of accurate data collection.

In addition to data accuracy, comprehensive reporting is important. Although genomics can be used to provide evaluations for animals without an observed trait, continued submission of phenotypic data still is needed to maintain accuracy. In recognition of the value of data, the CDCB makes payments to dairy records processing centers for providing data, supports the collection of feed efficiency data, and structures the fees for genomic evaluation to give a discount to data contributors.

The CDCB has a quality assurance program for the genotyping laboratories and nominators that includes monthly report cards and annual reviews. To become certified, the organizations must demonstrate the ability to provide data in the required formats. The monthly reports for labs include SNP accuracy and completeness as well as the percentage of genotypes with nomination and animal genotypes with a low call rate. The labs receive reports on those characteristics for each submission for possible correction before submissions are to added to the database.

## 4 Conclusion

The popularity of the genomic evaluation program in the United States has resulted in a rapid growth in the genotyping of dairy cattle. When genomic evaluation began in 2008, the focus was on bulls, but genotyping of females has grown rapidly in recent years. Many dairy producers genotype all their heifers so that they can select among a range of breeding and management strategies.

Dairy genetics in the United States and worldwide have been transformed by the use of genomic information. Genomic evaluations determine the value of animals at a much earlier age and have contributed to a dramatic increase in the rate of genetic improvement. Bulls are used widely as sires based on the analysis of their DNA before they have any milking daughters. A continuing stream of improvements are planned to increase accuracy and comprehensiveness of genomic evaluations. Success requires a partnership between data suppliers and users to generate the most effective information for all.
